# Optimising Vine Weevil, *Otiorhynchus sulcatus* F. (Coleoptera: Curculionidae), Monitoring Tool Design

**DOI:** 10.3390/insects13010080

**Published:** 2022-01-12

**Authors:** Eugenia Fezza, Joe M. Roberts, Toby J. A. Bruce, Lael E. Walsh, Michael T. Gaffney, Tom W. Pope

**Affiliations:** 1 Centre for Integrated Pest Management, Agriculture and Environment Department, Harper Adams University, Newport, Shropshire TF10 8NB, UK; jroberts@harper-adams.ac.uk (J.M.R.); tpope@harper-adams.ac.uk (T.W.P.); 2 Horticulture Development Department, Teagasc, Ashtown Research Centre, D15 DY05 Dublin, Ireland; Lael.Walsh@teagasc.ie (L.E.W.); Michael.Gaffney@teagasc.ie (M.T.G.); 3 Centre for Applied Entomology and Parasitology, School of Life Sciences, Huxley Building, Keele University, Staffordshire ST5 5BG, UK; t.j.a.bruce@keele.ac.uk

**Keywords:** horticulture, monitoring, integrated pest management, visual cues, Curculionidae

## Abstract

**Simple Summary:**

Vine weevil remains one of the most economically important insect pests of soft-fruit and ornamental crops globally. Growers currently lack effective monitoring systems to determine presence of vine weevil within crops, meaning that controls are often applied too late to prevent economic losses. Development of improved monitoring systems is currently hindered by a lack of knowledge of whether vine weevil adults select a monitoring tool based on its visual appearance. This study used paper cups as refuges to investigate the importance of colour, shape and position of entrances on monitoring tool efficacy. Results indicate that dark, tall refuges with entrances around their base were preferentially entered by adult vine weevil. This information provides the first steps towards developing improved designs for vine weevil monitoring tools.

**Abstract:**

Vine weevil, *Otiorhynchus sulcatus* F. (Coleoptera: Curculionidae), is an economically important insect pest of horticultural crops. To identify an effective and reliable monitoring system for adult vine weevil, this study investigated the influence of colour, height and entrance position on the efficacy of a model monitoring tool using modified paper cups as refuges. Vine weevil preferences were determined by the number of individuals recorded within a refuge. When provided with a binary choice between black or white refuges, vine weevil adults showed a preference for black refuges. Vine weevils provided with a range of coloured refuges (blue, green, red and yellow) in addition to black and white refuges showed a preference for black and blue over the other colours and white refuges in group choice experiments. Refuge height and entrance position also influenced vine weevil behaviour with individuals exhibiting a preference for taller refuges and those with entrance openings around the refuge base. These results provide insights into refuge selection by adult vine weevils, which can be exploited to improve monitoring tool design. The importance of developing an effective monitoring tool for vine weevil adults as part of an integrated pest management programme is discussed.

## 1. Introduction

Vine weevil, *Otiorhynchus sulcatus* (Fabricius) (Coleoptera: Curculionidae), is one of the most economically important pest species of soft-fruit and ornamental crops globally [1,2]. This polyphagous species has a Palearctic distribution but has been inadvertently introduced to several regions outside their native range through plant trade routes [1]. Populations of this species are now widespread across the USA, New Zealand, Chile and Japan while localised populations have been observed in Australia and Canada [3].

Adult vine weevils preferentially feed on plant foliage leading to characteristic ‘notching’ along the leaf perimeter [4]. While adult feeding does not significantly impact plant health, it can reduce the economic value of ornamental plants where there is a lower tolerance for aesthetic imperfections. A single individual can result in crops being rejected due to feeding damage and risk of further infestations due to eggs being laid around the plant base [5,6,7,8,9]. Larvae feed on belowground plant tissues such as roots, corms and rhizomes [1,10,11], which disrupts water and nutrient transport to aboveground plant tissues. This reduces vigour and water absorption leading to reduced plant growth, yield and survival compared to uninfested plants [1,7]. Growers have historically relied on broad-spectrum synthetic chemical insecticides for vine weevil control [10,12,13,14,15,16]. Many of these products have, however, been withdrawn from the market due to their negative impacts on the environment and human health [17,18,19]. With fewer synthetic chemical insecticide options available for vine weevil control, there has been a shift to using biological controls, such as entomopathogenic nematodes (EPNs) and fungi (EPFs) [20,21,22,23,24,25].

A core principle of integrated pest management (IPM) is to base the use of any control measures on pest population monitoring in relation to action thresholds [26,27,28]. Current methods for monitoring vine weevil adults include use of visual searches for the presence of leaf notching and shaking or tapping bushes to dislodge adults at night [29,30]. These approaches are, however, considered to be time consuming and unreliable. A cost-effective and reliable method for monitoring vine weevil adults at low population densities is needed to help growers make better informed vine weevil management decisions. One possible approach is the use of traps and artificial refuges as monitoring tools. Different monitoring tool designs have been used to detect the presence of vine weevil populations, including grooved wooden boards placed on the ground [29,31], pitfall traps [32], corrugated cardboard or ruffled material wrapped around plants [33,34] and commercially available crawling insect traps [25], including one designed specifically for vine weevil [35]. Despite extensive work to develop vine weevil monitoring tools, their efficacy is variable and often cannot be used to confidently monitor vine weevil populations in crops [35]. This lack of sensitivity and reliability reflects, in part, our limited understanding of the vine weevil adult visual ecology.

Insect vision is a fundamental consideration when developing monitoring tools [36,37,38,39]. Certain monitoring tool characteristics, such as colour and shape, are known to be key factors in increasing their efficacy [40,41]. For example, red palm weevil (*Rhynchophorus ferrugineus*) shows a preference for black traps over traps of different colours [42,43]. Leskey [38] showed that pyramid-shaped traps are more effective than cylindrical traps for monitoring plum curculio weevil (*Conotrachelus nenuphar*). Previous studies have also highlighted the importance of trap size, showing certain weevil species have a preference for taller silhouettes [44,45]. Furthermore, a direct relationship has been shown between the number of monitoring tool entrance holes and number of rice weevils (*Sitophilus oryzae*) captured [46].

For weevil species where research has been completed, visual cues are known to be important in the location of hosts and conspecifics [47,48,49]. Little research on the behavioural responses of adult vine weevil to visual stimuli has, however, been carried out to date. This study tests whether the behavioural responses of vine weevil adults towards prototype refuges under laboratory conditions are influenced by visual stimuli such as refuge colour, size and entrance location.

## 2. Materials and Methods

### 2.1. Insect Cultures

Vine weevil at various larval stages were collected from commercial strawberry (*Fragaria × ananassa* cv. Duchesne) crops grown in Shropshire and Staffordshire (UK) during autumn 2020. An equal number of larvae were collected from each site and combined into one laboratory culture to minimise potential bias during experiments due to sampling from multiple collection sites. Larvae were maintained on strawberry (*Fragaria × ananassa* cv. Elsanta) or primrose (*Primula vulgaris*) plants housed within a controlled environment room (20 °C; 60% relative humidity; 16:8 photoperiod) (Fitotron, Weiss Technik, Ebbw Vale, Wales, UK). Larvae that completed their development were maintained as adults under the previously stated environmental conditions using a standard method of placing the weevils in plastic terrariums (30 × 19.3 × 20.6 cm) containing yew (*Taxus baccata*) branches and moist paper towels that were replaced weekly [23,25,35]. All adult weevils used in the following experiments were at least one month old and had been confirmed to be reproductively active.

### 2.2. Refuge Colour (Dark vs. Light)

The behavioural response of adult vine weevils to light and dark coloured refuges was tested in a binary-choice experiment investigating individual and group selection under controlled environment conditions (20 °C; 60% relative humidity; 16:8 photoperiod). Refuges were created from paper cups (height = 7 cm; Ø base = 5 cm; Ø rim = 7 cm) that were externally and internally painted either black or white using poster paint (Galeria Acrylic, Windsor & Newton, London, UK). Paper cups used as refuges were inverted so that the rim became the refuge base and four equally distanced entrances were made in the refuges by cutting 1 cm^2^ openings around the cup rim. A roll of corrugated card (length = 30 cm; width = 3 cm) was inserted into each refuge to provide shelter.

**Figure 1:**
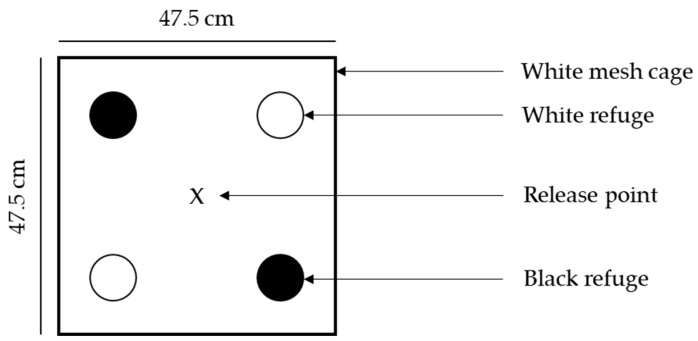
Schematic diagram showing the arrangement within each mesh cage for the refuge colour (dark vs. light) experiment. Refuge positions are shown in circles and the vine weevil release point using an x mark.

Five experimental arenas were prepared by placing four refuges (two black and two white) in the corners of five white mesh cages (47.5 × 47.5 × 47.5 cm, BugDorm-4S4545, MegaView Science Co. Ltd., Taichung, Taiwan) (Figure 1). Refuge positions were randomised between replicates to account for potential directional bias. A piece of damp cotton wool and yew leaves were placed in the centre of each cage to provide a source of food and moisture. For the experiment where one weevil adult was released a total of 50 individuals were tested (five replicates per day for ten days) while for the experiment where five vine weevil adults were released a total of 125 individuals were tested (five replicates per day for five days). As vine weevil are nocturnal and seek refuges as dawn approaches, individuals were released three hours prior to the controlled environment room lights switching off at 22:00. Controlled environment room lighting remained off until 06:00 and the position of each vine weevil was recorded at 07:00, providing a one-hour period for vine weevil to seek refuge before assessment began.

An additional experiment was carried out to determine the effect of background colour on vine weevil preference for black or white refuges. This experiment was carried out as previously described (testing both individual and group selection) but the cage floor and sides were covered with black paper (Clairefontaine Coloured Kraft Roll, ExaClair, King’s Lynn, UK).

### 2.3. Refuge Colour (Multiple)

The behavioural response of adult vine weevils to differently coloured refuges (blue, green, red or yellow), as well as black and white refuges, were tested in a six-choice experiment under the same controlled environment conditions as described previously. Refuges were created from paper cups (height = 7 cm; Ø base = 5 cm; Ø rim = 7 cm) and painted one of four colours or black or white using poster paint. Refuge colour was determined using reflectance measurements acquired using a spectrophotometer (Flame Miniature Spectrometer, Ocean Insight, Duiven, The Netherlands) with a spectral wavelength range from 450 to 700 nm (see Supplementary Figure S1. Paper cups were inverted as previously described and four equally distanced entrances created around the cup rim to create refuges. A roll of corrugated card (length = 30 cm; width = 3 cm) was inserted into each refuge to provide shelter. Two experimental arenas were prepared depending on the number of vine weevil adults being released (either one or ten). Refuges were positioned 15 cm from one another in a hexagon centrally positioned within a white mesh cage (47.5 × 47.5 × 47.5 cm) for replicates using one vine weevil adult while refuges were positioned 20 cm from one another in a hexagon centrally positioned within a larger white mesh cage (57.5 × 57.5 × 57.5 cm; MegaView Science Co., Ltd., Taichung, Taiwan) for replicates using ten vine weevil adults. Each refuge position was randomly altered between replicates to account for potential directional bias. A piece of damp cotton wool and yew leaves were placed in the centre of each cage to provide a source of food and moisture. A total of 50 individuals were tested (five replicates per day for ten days) releasing a single vine weevil adult, while 200 individuals were tested (four replicates per day for five days) for replicates releasing ten vine weevil adults. The timing of weevil release and assessment after lights had come back on was the same as previously described.

### 2.4. Refuge Height

The behavioural response of adult vine weevil to differently sized refuges was tested in a three-choice experiment under the same controlled environment conditions as described previously. Refuges were modified from paper cups (height = 11.3 cm; Ø base = 5.8 cm; Ø rim = 9.8 cm) by removing the cup base and sides to create three heights: 11.3 cm, 6 cm and 3 cm. For the cups to be used as refuges they were inverted so that the rim became the refuge base and four equally distanced entrances created around the rim. As the cups were inverted to create refuges, cup rims were left unaltered, except for inclusion of entrances, to ensure bases were consistent between treatments (Ø rim = 9.8 cm).

Refuges were painted externally and internally black using poster paint. A roll of corrugated card (length = 30 cm; width = 3 cm) was inserted into each refuge to provide shelter as for the previous experiments. Experimental arenas were prepared by placing three refuges (one of each height) in a triangle 30 cm from one another inside a white mesh cage (47.5 × 47.5 × 47.4 cm). Each refuge position was randomly altered between replicates to account for potential directional bias. A piece of damp cotton wool and yew leaves were placed in the centre of each cage to provide a source of food and moisture. Ten adult vine weevils were released from the centre of each cage. A total of 200 individuals were tested (four replicates per day for five days) during this experiment. The timing of weevil release and assessment after lights had come back on was the same as previously described.

### 2.5. Refuge Entrance Position

The behavioural response of adult vine weevil to refuges with differing entrance positions was tested in a four-choice experiment under the controlled environment conditions as described previously. All refuges were modified from paper cups (height = 11.3 cm; Ø base = 5.8 cm; Ø rim = 9.8 cm) to create four different entrance configurations: (1) four 1 cm^2^ entrances equally distanced from one another around the refuge base, (2) one 1 cm high continuous entrance around the refuge base, (3) four 1 cm^2^ entrances equally distanced from one another 8.3 cm from the refuge base and (4) four 1 cm^2^ entrances equally distanced from one another at the top of the refuge. Refuges were painted externally and internally black using poster paint. Experimental arenas were prepared placing by four refuges (one of each entrance configuration) in the corners of a 30 cm^2^ square centrally positioned within a white mesh cage (47.5 × 47.5 × 47.5 cm). Each refuge position was randomly altered between replicates to account for potential directional bias. A piece of damp cotton wool and yew leaves were placed in the centre of each cage to provide a source of food and moisture. Ten adult vine weevils were released from the centre of each cage. A total of 200 individuals were tested (four replicates per day for five days) during this experiment. The timing of weevil release and assessment after lights had come back on was the same as previously described.

### 2.6. Statistical Analysis

All statistical analyses were carried out using R (Version 3.6.2) [50]. Refuge performance (i.e., the number of individuals within refuge) for binary-choice experiments was analysed using an exact binomial test against the null hypothesis that the number of vine weevils seeking refuge had a 50:50 distribution. Prior to carrying out statistical analyses, replicated results from each of the refuge designs tested were pooled. All other experiments were analysed using generalised linear models (GLMs) fitted with Poisson probability distributions. Multiple comparisons for Poisson probability distribution data were evaluated by Tukey’s HSD tests implemented in the *HSD.test* function in the R package *agricolae* [51]. Individuals not recorded in the refuges were excluded from statistical analysis.

## 3. Results

### 3.1. Refuge Colour (Dark vs. Light)

**Figure 2:**
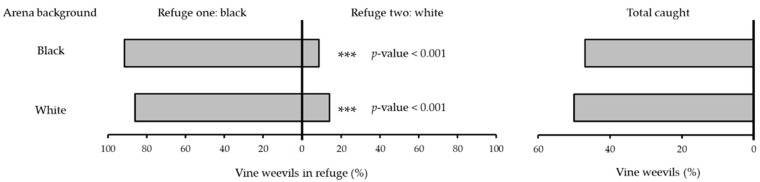
Percentage of vine weevil adults recorded in refuges painted black or white when released individually (*number of replicate days* = 10) into a cage with a black background or white background. Asterisks indicates significant differences between refuge choices.

**Figure 3:**
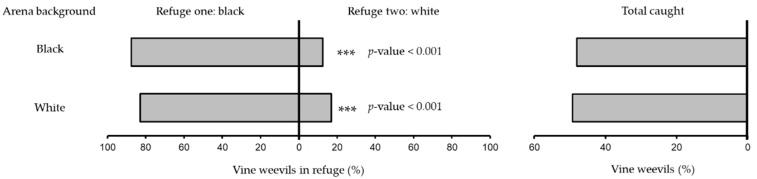
Percentage of vine weevil adults recorded in refuges painted black or white when released as a group of five individuals (*number of replicate days* = 5) into a cage with a black background or white background. Asterisks indicates significant differences between refuge choices.

In binary-choice experiments presenting adult vine weevil with black and white refuges against a black background, the number of individuals recorded in black refuges was significantly higher than in white refuges in both single (binomial exact test: no. successes = 43, no. trials = 47, *p* < 0.001) (Figure 2) and group release experiments (binomial exact test: no. successes = 105, no. trials = 125, *p* < 0.001) (Figure 3) with 91% and 88%, respectively. When the choice between black and white refuges was presented against a white background significantly more vine weevil were recorded in black refuges than white refuges in both individual (binomial exact test: no. successes = 47, no. trials = 50, *p* < 0.001) (Figure 2) and group release experiments (binomial exact test: no. successes = 102, no. trials = 123, *p* < 0.001) (Figure 3) with 86% and 83%, respectively.

### 3.2. Refuge Colour (Multiple)

**Figure 4:**
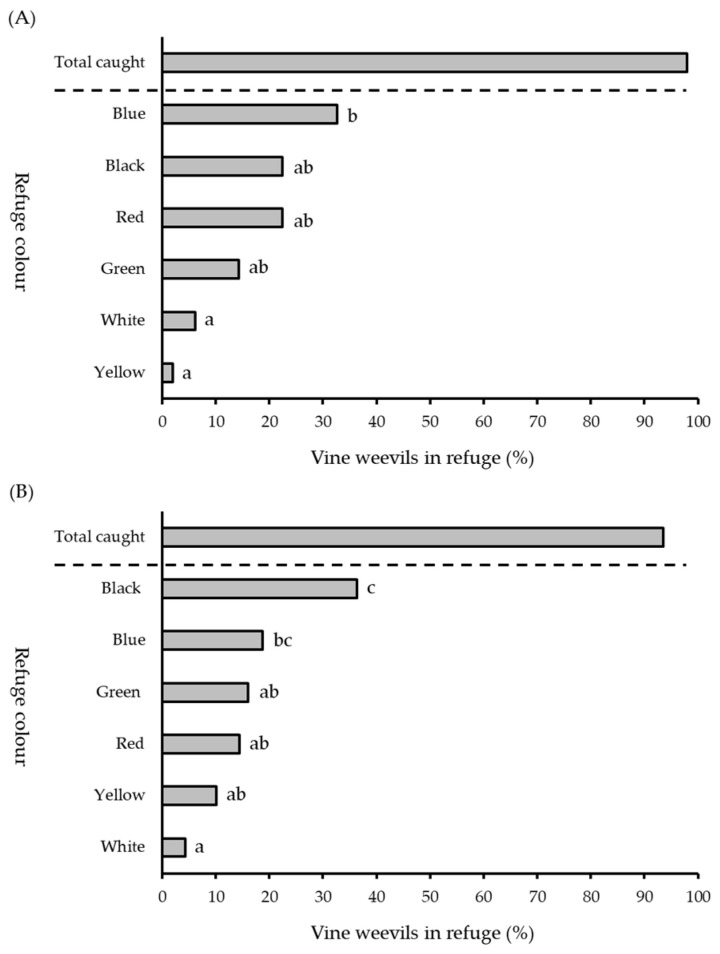
Percentage of vine weevil adults recorded in refuges painted blue, green, red, yellow, black or white when released (**A**) individually (*number of replicate days* = 10) or (**B**) as a group of ten individuals (*number of replicate days* = 5). Different letters denote significant differences between means (*p* < 0.05).

In six-choice experiments presenting adult vine weevil with black, white, blue, green, red and yellow refuges against a white background there was a significant difference in individuals recorded in refuges for both single (generalised linear model: *F*^2^ = 241.07, *df* = 294, *p* < 0.05) and group release experiments (generalised linear model: *F*^2^ = 157.47, *df* = 114, *p* < 0.05) (Figure 4). The percentage of adult vine weevil recorded in blue (32.65%), black (22.45%), red (22.45%) and green (14.28%) (Figure 4A) refuges was greater than white (6.12%) or yellow (2.04%) refuges when adult vine weevil were released individually (Tukey’s HSD test: *p* < 0.05, see Supplementary Table S1. Black refuges contained the largest number of adult vine weevil when released in groups (36.36%) (Figure 4B) though this was not significantly greater than blue (18.72%) refuges (Tukey’s HSD test: *p* > 0.05, see Supplementary Table S2.

### 3.3. Refuge Height

**Figure 5:**
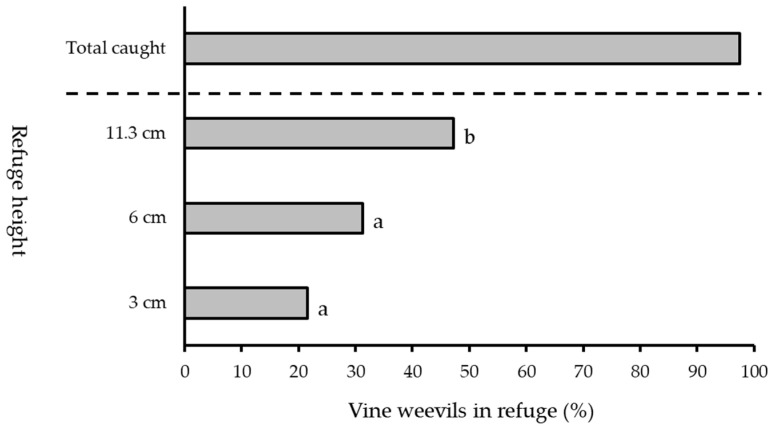
Percentage of vine weevil adults recorded in refuges with a height of 3, 6 or 11.30 cm when released as a group of ten individuals (*number of replicate days* = 5). Different letters denote significant differences between means (*p* < 0.05).

In three-choice experiments presenting adult vine weevil with three refuges of varying heights against a white background, height had a significant effect on the number of weevil adults recorded in the refuge (generalised linear model: *F*^2^ = 52.09, *df* = 57, *p* < 0.05) (Figure 5). A higher percentage of adult vine weevils were recorded in the taller refuges (11.30 cm; 47%) than the medium (6 cm; 31%) and short (3 cm; 22%) refuges (Tukey’s HSD test: *p* < 0.05, see Supplementary Table S3.

### 3.4. Refuge Entrance Positions

**Figure 6:**
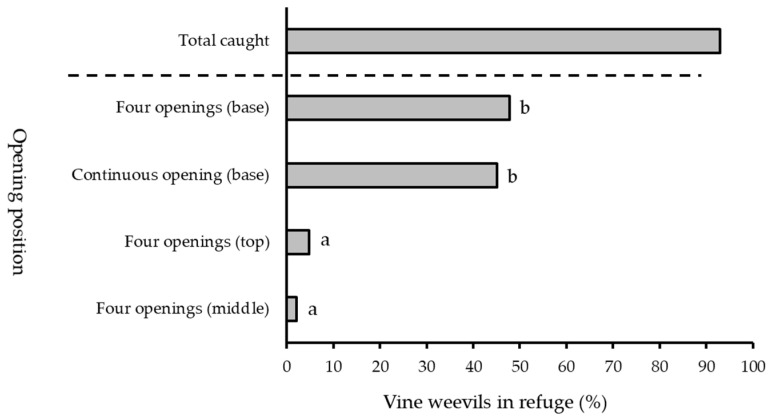
Percentage of vine weevil adults recorded in refuges with openings on the side, top or base—continuous opening or four openings—when released as a group of ten individuals (*number of replicate days* = 5). Different letters denote significant differences between means (*p* < 0.05).

In four-choice experiments presenting adult vine weevil with four refuges that had varying entrance configurations against a white background, entrance position had a significant effect on the number of individuals recorded in the refuges (generalised linear model: *F*^2^ = 62.19, *df* = 76, *p* < 0.05) (Figure 6). Refuges with entrances positioned around their base contained higher percentages of adult vine weevil (48% in refuges with four entrances; 45% in refuges with a continuous entrance) compared to those with entrances at the top (5%) or in the middle (2%) (Tukey’s HSD test: *p* < 0.05, see Supplementary Table S4.

## 4. Discussion

Phytophagous insects use a wide range of environmental cues to locate potential host plants within a heterogeneous environment [52,53]. A greater understanding of pest insect visual ecology can inform monitoring tool development to improve their efficacy and increase grower confidence in using them [54,55,56,57,58,59]. This study recorded adult vine weevil behavioural responses to prototype monitoring tool designs and sought to determine which factors influence selection of a refuge. Results presented here indicate that adult vine weevil, released either individually or as groups, prefer dark refuges (black) over light refuges (white) in two-choice experiments. While no comparable studies have been carried out with vine weevils, these results support studies carried out with the palm weevil where black traps caught more individuals than white traps [41,42,43,60].

Differences in luminance or coloration due to surface patterns or shadows may be perceived as contrast signals. Insects use contrast to discriminate objects from their background and to recognise shapes [36,61,62]. Entwistle [63] and Timmons and Potter [64] speculated that insects caught by red, brown and black traps are likely responding to dark shades and the contrast with the background rather than to any visual cue. Our study contradicts this hypothesis as differences between background colour, and therefore refuge contrast, did not influence adult vine weevil behaviour. This result will require further investigation to fully understand how vine weevil adults perceive objects in different environments.

Several studies have shown that monitoring tool efficacy can be impacted by colour [57,58,59], indicating that colour plays an important role in insect behavioural responses to such tools. When released individually or as small groups, vine weevil adults preferred dark refuges (black and blue) over light refuges (white and yellow) in six-choice experiments. It is notable that a nocturnal species, such as vine weevil, could be influenced by colour as they are active when colour is most challenging to perceive. However, colour preference has been demonstrated for several nocturnal insect species [45]. Tree-of-heaven root weevil (*Eucryptorrhynchus scrobiculatus*) adults exhibit a preference for red and black traps over yellow traps [65], while pales weevil (*Hylobius pales*) and the pitch-eating weevil (*Pachylobius picivorus*) prefer black or brown traps over yellow traps [37]. Variation among weevil species in their colour preference may depend on the resemblance to suitable habitats for mating, oviposition or feeding. For example, many herbivorous insects respond to yellow or white as it corresponds to peak host plant colour reflectance [36,41]. Pales weevil and pitch-eating weevil are root feeding weevils attracted to black and brown over other colours because these resemble root environments [37]. Bark beetles respond to any colour presenting dark silhouettes for their similarity to conifer host tree trunks and traps that provide appropriate visual silhouettes are more effective [36,66,67].

There are a range of other factors that may influence monitoring tool efficacy. Silva et al. [41] showed that boll weevil traps, designed to mimic the colour of foliage, were less effective when used for cranberry weevil (*Anthonomus musculus*) adults than sticky traps of similar colour. This suggests that trap design, and not only colour, is important for cranberry weevil. Shape is known to be a key factor influencing insect monitoring tool efficacy [48,68,69]. Refuge height impacts the refuge shape and has been shown to directly influence the behaviour of target organisms. Tree-of-heaven root weevil and walking white pine weevil (*Pissodes strobi*) adults orientated preferentially towards silhouettes that are taller rather than shorter [65,70]. Taller refuges contained more individuals than shorter refuges in our study, suggesting that this may also be an important factor influencing adult vine weevil behaviour. Having taller refuges may also improve monitoring tool sensitivity in a similar fashion to wider refuges as they have a larger surface area, providing more shelter for weevils [45]. Although undoubtedly important, there are other factors beyond shape and size that influence monitoring tool efficacy. For example, Roberts et al. [35] found that despite the general design (colour and silhouette) of vine weevil and red palm weevil traps being similar, their efficacy differed due to other characteristics including the entrance position. Our results demonstrated that vine weevil prefer to enter refuges with openings at their base over those with entrances at the top or in the middle. Although many studies have assessed the effect of monitoring tool design on efficacy, to our knowledge no studies have specifically examined the effect of entrance position and have instead focused on the effect of entrance number and spacing [46,71,72,73].

## 5. Conclusions

Currently available vine weevil monitoring tools have variable efficacy and do not reliably indicate pest presence or density under field conditions [34]. A better understanding of vine weevil visual ecology and how they interact with monitoring tools will help inform their optimisation and improve detection and monitoring of this pest [35]. Trap location, size and surface texture are reported to influence weevil monitoring efforts [49,74,75]. This study provides evidence of the importance of visual ecology in the development of effective and reliable vine weevil monitoring tools and indicates that tall dark shapes are likely to be most effective for this species. However, interactions between visual and olfactory signals are still to be investigated; in particular, how blends of VOCs based on host plants interact with the visual appearance of a monitoring tool.

While the results presented here provide a first step toward understanding the visual preferences of adult vine weevil, it is important to remember that insects may perceive colour differently in outdoor and indoor conditions [76,77] and so further in-field testing of these visual preferences is required.

## Data Availability

The datasets used and/or analysed during the current study are available from the corresponding author on request.

## Supplementary Materials

The following are available online at https://www.mdpi.com/article/10.3390/insects13010080/s1, Figure S1: Reflectance values from spectral analysis of coloured paper cups used in trapping experiments. Table S1, Tukey contrast comparison between painted refuges when vine weevil adults were released individually. Table S2: Tukey contrast comparison between painted refuges when vine weevil adults were released in groups of 10. Table S3: Tukey contrast comparison between refuges with different heights. Table S4: Tukey contrast comparison between refuge with different entrance configurations.
