# Supplementary material for: Optimising Vine Weevil, Otiorhynchus sulcatus F. (Coleoptera: Curculionidae), Monitoring Tool Design
Source: Insects. 2022 Jan 12;13(1):80. doi: 10.3390/insects13010080 (PMC8777626; doi:10.3390/insects13010080)
Supplement: Supplementary file 1 [file insects-13-00080-s001.zip › insects-1518082-supplementary.pdf]

Supplementary Materials

Refuge colour measurements

The painted paper cups used as refuges in this study had differences in reflectance along the visible spectrum (450–700 nm) (Supplementary figure 1). White refuges had high reflectance (90 %) at wavelengths above ~ 550 nm. In contrast, black refuges had zero or close to zero reflectance at the wavelengths measured. Yellow refuges also had high reflectance (80 %) above ~ 560 nm. The reflectance of blue refuges was 40 % between ~ 455 and 475 nm, while green refuges had a maximum spectral reflectance (50 %) between ~ 495 and 510 nm. Red refuges had high reflectance (70 %) at wavelengths above ~ 625 nm.

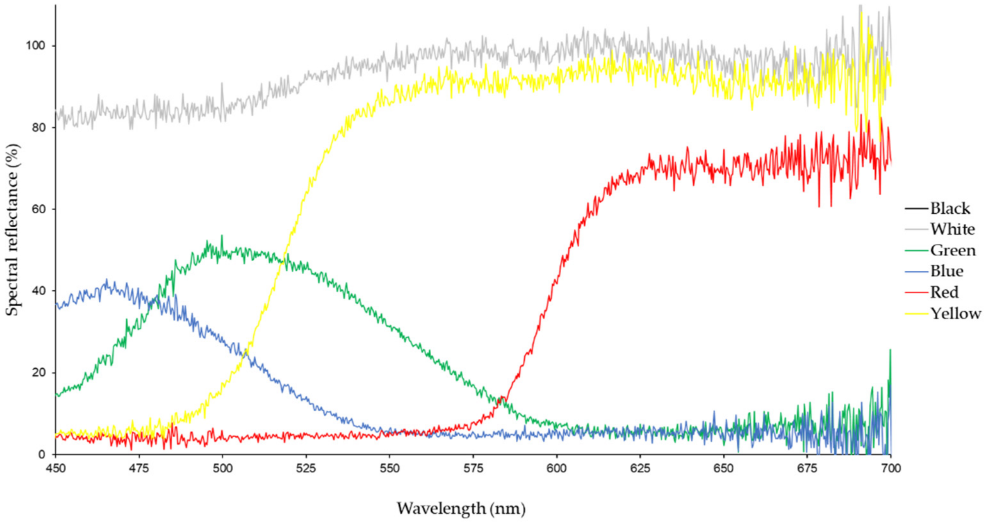

Supplementary Figure S1. Reflectance values from spectral analysis of coloured paper cups used in trapping experiments.

Supplementary Table S1. Tukey contrast comparison between coloured refuges when vine weevil adults were released individually

Comparison	Odds ratio	SE	p. value
White / Yellow	3.13	3.67	0.93
Green / Yellow	7.98	8.69	0.398
Green / White	2.55	1.84	0.79
Black / Yellow	13.82	14.74	0.13
Black / White	4.42	3.03	0.25
Black / Green	1.73	0.92	0.91
Red / Yellow	13.82	14.74	0.13
Red / White	4.42	3.03	0.25
Red / Green	1.73	0.92	0.91
Red / Black	1.00	0.48	1.00
Blue / Yellow	23.06	24.31	0.03
Blue / White	7.37	4.93	0.03
Blue / Green	2.89	1.47	0.29
Blue / Black	1.67	0.76	0.87
Blue / Red	1.67	0.76	0.87

Supplementary Table S2. Tukey contrast comparison between coloured refuges when vine weevil adults were released in groups of 10

Comparison	Odds ratio	SE	p. value
Blue / Black	0.51	0.12	0.05
Green / Black	0.44	0.11	0.01
Green / Blue	0.86	0.24	0.99
Red / Black	0.39	0.1	0.004
Red / Blue	0.77	0.22	0.95
Red / Green	0.9	0.27	0.99
White / Black	0.12	0.049	<.0001
White / Blue	0.23	0.1	0.01
White / Green	0.27	0.12	0.05
White / Red	0.29	0.13	0.08
Yellow / Black	0.28	0.08	0.0002
Yellow / Blue	0.54	0.17	0.4

Yellow / Green	0.63	0.21	0.74
Yellow / Red	0.7	0.24	0.9
Yellow / White	2.37	1.13	0.46

Supplementary Table S3. Tukey contrast comparison between refuges with different heights

**Comparison**	**Odds ratio**	**SE**	**p. value**
3 cm / 11.30 cm	0.46	0.08	<.0001
6 cm / 11.30 cm	0.66	0.1	0.02
6 cm / 3 cm	1.45	0.28	0.12

Supplementary Table S4. Tukey contrast comparison between refuge with different entrance configurations

**Comparison**	**Odds ratio**	**SE**	**p. value**
continuous opening (base)/ four openings (base)	0.94	0.14	0.98
four openings (side)/ four openings (base)	0.04	0.02	<.0001
four openings (side)/continuous opening (base)	0.05	0.02	<.0001
four openings (top)/ four openings (base)	0.1	0.03	<.0001
four openings (top)/continuos opening (base)	0.11	0.04	<.0001
four openings (top)/ four openings (side)	2.25	1.35	0.53
